# Relationship Between Sleep Disturbances and Chronic Pain: A Narrative Review

**DOI:** 10.3390/clinpract14060209

**Published:** 2024-12-09

**Authors:** Sejal V. Jain, Geoffrey D. Panjeton, Yuri Chaves Martins

**Affiliations:** 1 Department of Anesthesiology and Pain Management, The University of Texas Southwestern Medical Center, Dallas, TX 75390, USA; sejal.jain@utsouthwestern.edu; 2 Department of Anesthesiology, Saint Louis University School of Medicine, St. Louis, MO 63104, USA; geoffrey.panjeton@health.slu.edu

**Keywords:** bidirectional relationship, chronic pain, polysomnography, sleep disorders, sleep disturbances

## Abstract

Sleep disturbances and chronic pain are prevalent and interrelated conditions that have significant impact on individuals’ quality of life. Understanding the intricate dynamics between sleep and pain is crucial for developing effective treatments that enhance the well-being of affected individuals and reduce the economic burden of these debilitating conditions. This narrative review examines the complex relationship between sleep disturbances and chronic pain. We describe the prevalence and types of sleep disturbances and sleep disorders in chronic pain patients. Posteriorly, we critically review the clinical and experimental evidence, investigating the relationship between sleep disturbances and chronic pain, aiming to clarify the impact of chronic pain on sleep and, conversely, the impact of sleep disturbances on pain perception. In conclusion, the literature largely agrees on the existence of a bidirectional relationship between chronic pain and sleep disturbances, though the strength of each direction in this association remains uncertain. Current evidence suggests that sleep impairment more strongly predicts pain than pain does sleep impairment. Additionally, addressing sleep disturbances in chronic pain patients is crucial, as poor sleep has been linked to higher levels of disability, depression, and pain-related catastrophizing.

## 1. Introduction

Human sleep is a naturally recurring state of mind and body characterized by altered consciousness, relatively inhibited sensory activity, reduced muscle activity, inhibition of nearly all voluntary muscles during rapid eye movement (REM) sleep, and reduced interactions with surroundings [1]. It is a vital, restorative process that allows the body and mind to maintain homeostasis, develop, and optimize function across multiple physiologic systems [1,2]. Sleep disturbances encompass a range of disorders that disrupt the normal sleep cycle, including insomnia, narcolepsy, sleep apnea, restless legs syndrome (RLS), and circadian rhythm disorders [3,4]. The prevalence of sleep disturbances in the general population varies widely across different populations and conditions [3,4,5,6,7]. For instance, the prevalence of sleep-disordered breathing (SDB) ranges from 9.0 to 83.3% in men and from 4.0 to 76.6% in women [3]. Insomnia affects approximately 22% [7], narcolepsy affects 0.04–0.1% [4,6], and obstructive sleep apnea (OSA) impacts from 9 to 38% of the adult population [5]. Sleep disturbances are associated with a myriad of negative health outcomes including cognitive dysfunction, immune dysregulation, and increased risk of cardiovascular disease, diabetes, and obesity [8,9].

Pain is a complex and multifaceted phenomenon defined as an unpleasant sensory and emotional experience associated with actual or potential tissue damage or described in terms of such damage [10]. Chronic pain is defined as pain that persists or recurs for more than three months [11]. Chronic pain often lasts beyond the usual course of an acute illness or healing of an injury and can continue despite the absence of an obvious injury or disease [11]. The prevalence of chronic pain in the adult population is estimated to be between 19.2 and 41.4% with rates up to 76.2% in the elderly [12,13,14]. The incidence of chronic pain and severe chronic pain in the USA was recently estimated as 52.4 and 12.0 cases per 1000 person-years, respectively [15]. This incidence is greater than other common chronic conditions such as diabetes, hypertension, and depression [15]. The economic burden of chronic pain is substantial, encompassing healthcare costs, lost work productivity, and decreased quality of life [16]. As with sleep disturbances, chronic pain is also associated with similar negative health outcomes including cardiovascular diseases, cerebrovascular events, cognitive impairment, psychiatric diseases, and disability [2,17].

Consequently, sleep disturbances and chronic pain are prevalent health issues that can independently impact individuals’ quality of life and overall well-being. However, clinical and experimental studies have also consistently shown that sleep disturbances and chronic pain have a complex relationship that may exacerbate the severity and impact of each other [18,19,20]. In the present work, we critically review the clinical and experimental evidence investigating the relationship between sleep disturbances and chronic pain, aiming to better answer some fundamental questions that have been puzzling the field since its inception: (1) What are the prevalence and types of sleep disturbances and sleep disorders in chronic pain patients?; (2) What is the impact of chronic pain on sleep?; and, (3) What is the impact of sleep disturbances on pain perception? The answers to these questions are of paramount importance, as unraveling the complexities of the interaction between sleep and pain can allow the development of more effective approaches to improve the health and well-being of those affected by these debilitating conditions and potentially significantly decrease the economic burden caused by these diseases.

## 2. Methods

We formulated the three primary questions to guide this review based on our clinical experience and expertise in managing chronic pain patients. These questions were defined prior to initiating the literature search to ensure a focused and systematic approach. A comprehensive literature search was then conducted across PubMed, Google Scholar, Embase, and Web of Science for studies published from 2010 onward. Keywords used in the search were ‘sleep disturbance’, ‘sleep disorder’, ‘chronic pain’, and ‘sleep and pain interaction’.

Articles were included if they investigated the relationship between chronic pain and sleep disturbances in clinical populations. Reference tracking and citation searches were performed for papers published before 2010 only if they were considered seminal to answering the devised questions based on the analyses of more recent studies. Following duplicate removal, studies were screened by title and abstract for their potential to address our guiding questions. Selected studies were then reviewed in full to confirm relevance to the review’s objectives. Data extraction was performed to capture information on population characteristics, interventions, outcomes, and other relevant variables related to the interaction between chronic pain and sleep disturbances.

Findings were thematically organized, and a critical analysis was undertaken to identify key trends, areas of consensus, and limitations within the literature. Only peer-reviewed papers published in English were included.

## 3. Prevalence and Types of Sleep Disturbances and Sleep Disorders in Chronic Pain Patients

There is no clear definition of the term sleep disturbance in the literature [19,21,22,23]. In general, sleep disturbance refers to any disruption in the quality, quantity, or timing of sleep, and many times is suggestive of insomnia in a broad sense. It can be occasional or frequent and might not necessarily indicate a medical condition. On the other hand, sleep disorders are specific medical conditions that consistently impair the ability to sleep well on a regular basis. They are typically diagnosed based on established criteria and often require medical or psychological intervention.

Some studies have explored the prevalence and types of sleep disturbances in patients with chronic pain [2,18,19,24,25] (Table 1). An increased prevalence of sleep disturbances when compared with the general population was found in patients with different chronic pain syndromes, including chronic low back pain [26,27], orofacial pain [28], chronic joint pain [29], cancer-related pain [30,31,32], headaches [33], and fibromyalgia [25]. Therefore, in general, individuals with chronic pain have a higher risk to present or develop sleep disturbances [2,18,19,24]. Furthermore, chronic pain patients with concurrent sleep disturbances are more likely to have greater disability, psychological distress, depression, catastrophizing, anxiety, suicidal ideation, and be less physically active [20,22,34].

The prevalence of sleep disturbances among chronic pain patients varies significantly across different study populations, with rates reported between 40% and 88% [18,19,23]. The significant variability in prevalence estimates across studies may stem from differences in sample size, study design, and methodologies such as objective vs. subjective sleep assessments [2]. Objective sleep assessment with polysomnography is generally regarded as the ‘gold standard’, as opposed to self-reported measures, which are prone to inaccurate recall, the common expectation that pain disrupts sleep, and the high likelihood of memory and response biases [2,23]. Additionally, the predisposition to develop sleep disorders may differ based on the type of chronic pain syndrome the patient experiences. For instance, musculoskeletal conditions are often linked to sleep issues, with prevalence rates reaching up to 65% in rheumatoid arthritis, 70% in osteoarthritis, and 95% in fibromyalgia [19].

Insomnia appears to be the most common sleep disorder in chronic pain patients, with a prevalence ranging from 24 to 72% [22,23]. Restless legs syndrome (20–65.7%) and sleep-disordered breathing (10–83%) appear to come closely together in second [22,23]. It is unclear if other sleep disorders such as narcolepsy and parasomnias are more prevalent in patients with chronic pain when compared with the general population.

## 4. The Impact of Sleep Disturbances on Pain Perception

Sleep-deprived healthy subjects, in particular slow wave sleep restriction, show increased self-reported musculoskeletal pain, fatigue, and evoked pain responses obtained through somatosensory testing protocols (e.g., heat pain thresholds, pressure pain thresholds, or laser-evoked pain) when compared with healthy control subjects [2,35,36,37] (Table 2). There is also evidence showing that sleep deprivation produces or increases hyperalgesia in patients with acute pain and chronic pain [29,38,39,40]. Conversely, studies show improvement in pain threshold after improvement of sleep quantity or quality [41,42]. It is suggested, however, that findings from experimentally induced sleep disturbances may not fully replicate the experience of repeatedly waking throughout the night over extended periods, nor the long-term reduction in sleep quality commonly seen in chronic pain patients [43].

Prospective longitudinal studies examining the impact of sleep on future pain in both healthy individuals and chronic pain patients have aimed to address this issue and reported consistent findings. A population-based prospective study, which followed pain-free individuals for over 5 and 18 years, found that difficulties with sleep initiation, sleep maintenance, early awakening, and non-restorative sleep predicted the development of chronic widespread pain [44]. Pre-operative sleep disturbances are associated with increased post-operative acute surgical pain [38,39,40] and are a risk factor for chronic post-surgical pain [45]. Insomnia has been shown to be a risk factor for the development of musculoskeletal pain, back pain, headache, and osteoarthritic pain [26,46,47,48,49]. Patients with sleep-disordered breathing, narcolepsy, and sleep bruxism also have an increased prevalence of chronic pain [21,22]. Like insomnia, there is some evidence that narcolepsy is a risk factor for musculoskeletal pain, chronic low back pain, migraines, and tension-type headaches [50,51,52]. However, studies investigating the temporal relationship between sleep-disordered breathing and chronic pain are rare [21]. Consequently, there is no strong evidence that sleep-disordered breathing is a predictive factor for chronic pain development. Sleep disturbances can also increase pain intensity, pain sensitivity, and duration of pain in chronic pain patients [2,24,25,53,54]. Furthermore, sleep disturbances may contribute to the maintenance of symptoms in patients with chronic pain [55,56,57].

**Table 2:** Studies analyzing the impact of sleep disturbances on pain perception.

Study	Design	Measurements	Sample	Level of Evidence
Raymond et al. [39]	Cohort	VAS, hours of sleep, number of awakenings, report of nightmares	28 participants	Low
Varallo et al. [45]	Systematic review and meta-analysis	PSQI, VAS, PROMIS Profile-29- Sleep Problems subscale, PROMIS short form- Sleep Disturbance subscale, BPI, NRS	18 studies totaling 8408 participants with post-surgical pain	High
Aili et al. [44]	Cohort	USI, standardized questionnaire to diagnose CWP	1249 participants	Moderate
Orbach-Zinger et al. [38]	Cohort	PSQI, VNPS	245 pregnant women	Moderate
Khalid et al. [41]	Experimental	Polysomnography, ESS, FWL testing	12 participants	High
Roehrs et al. [42]	Experimental	Polysomnography, MSLT, sleep diary, pain tolerance thresholds to radiant heat	18 participants	High
Wang et al. [40]	Cohort	PSQI, NRS	108 women with breast cancer	Moderate
Smith et al. [36]	Experimental	PSG, sleep diaries, PILL, PPTh, cold pressor pain rating, DNIC index	32 women	High
Mohamed et al. [58]	Systematic review and meta-analysis	PSG, clinical diagnostic criteria	24 studies totaling 1278 participants	Moderate

BDI = The Beck Depression Inventory; BPI = Brief Pain Inventory; CATS = Pain Catastrophizing Scale; CES-D = Center for Epidemiologic Studies Depression Scale; CWP = Chronic Widespread Pain; DNIC = Diffuse Noxious Inhibitory Controls; ESS = Epworth Sleepiness Scale; FWL = Finger Withdrawal Latency Testing to a Radiant Heat Stimulus; HIT-6 = Headache Impact Test-6; ISI = Insomnia Severity Index; LBP = Low Back Pain; MIDAS = The Migraine Disability Assessment; MSLT = Multiple Sleep Latency Test; MPQ = McGill Pain Questionnaire-Short Form; NRS = Numerical Rating Scale; PANAS = Positive and Negative Affect Schedule; PILL = Pennebaker Inventory of Limbic Languidness; PPTh = Pressure Pain Threshold; PSG = Polysomnography; PSQ-3 = Pain and Sleep Questionnaire-3; PSQI = Pittsburgh Sleep Quality Index; STAI = State-Trait Anxiety Inventory; USI = Uppsala Sleep Inventory; VAS = Visual Analog Pain Intensity Scale; VNPS = Verbal Numerical Pain Score; VSH = Verran Snyder-Halpern Sleep Scale.

Studies also report that the treatment of sleep disturbances and disorders can improve chronic pain [2,59,60,61]. For example, sleep improvement by cognitive behavioral therapy predicts long-term pain improvement in patients with comorbid osteoarthritis and insomnia [62]. Similarly, chronic low back pain patients whose sleep disturbances resolved by follow-up were more likely to report recovery and lower pain intensities after six months [63]. Several other studies show improved pain after treatment of insomnia with sodium oxybate in patients with fibromyalgia and with melatonin in patients with temporomandibular joint pain [22]. However, some studies showed conflicting results with no change in pain outcomes despite improvement in sleep in patients with TMD and fibromyalgia treated with triazolam and zopiclone, respectively [22]. Additionally, studies show that treatment of OSA with CPAP improves experimental pain threshold [41,64]. Similarly, treatment of OSA with CPAP is associated with improvement in pain in patients with migraines and morning headaches [65,66]. Additionally, 78% of CPAP-adherent patients had headache improvements compared to 42% who did not try CPAP [65,66]. Contrary to these studies, Johnson et al. suggested that CPAP use at 12 months was not associated with any change in pain intensity in veterans [67].

Taken together this evidence indicates that sleep disturbances and sleep disorders can trigger or exacerbate pain intensity and functional impairment in chronic pain patients [21,56,68].

## 5. The Impact of Chronic Pain on Sleep

Studies analyzing the impact of chronic pain on sleep are less definitive than the ones analyzing the impact of sleep disturbances on chronic pain [2,21,22,23,34] (Table 3). Some studies have assessed sleep via polysomnography in acute post-surgical pain conditions [69]. Post-surgical patients show a reduction in total sleep time of up to 80%, decreased or absent REM sleep, and experience fragmented sleep with frequent arousals and awakenings during the first two nights after surgery [70,71,72,73]. They also show reduced duration of slow wave sleep for up to four nights [73]. While sleep is clearly disrupted in the post-operative period, determining the causal role as post-surgical pain is extremely difficult. Several factors may contribute to post-operative sleep disturbance, including hospital-related environmental factors, the stress response to the surgical insult, and medications used during the post-operative recovery [69].

Sleep disturbances found in patients with chronic pain include longer sleep-onset latency, more frequent and longer awakenings after sleep onset, unrefreshing sleep, shorter total sleep time, lower sleep efficiency, and poorer sleep quality [28,34,74,75]. This indicates that patients with chronic pain have less sleep time, take longer to fall asleep, and spend more time awake. Additionally, a recent meta-analysis found that chronic patients spent more time in the first stage of sleep during the non-NREM phase [23].

Most of the polysomnographic studies showing alterations during sleep in chronic pain were performed on fibromyalgia, arthritis, and chronic fatigue syndrome patients [23,76]. Polysomnography shows a reduction in the amount of slow wave sleep, REM sleep, and total sleep time associated with an increase in the number of arousals in patients with fibromyalgia when compared to age-matched healthy patients [77,78]. In addition, patients with arthritis and chronic fatigue syndrome exhibit alpha intrusions into slow wave and non-NREM sleep that are correlated with objective measurements of pain [29,76]. In contrast, there is conflicting data regarding alpha intrusions in sleep in patients with fibromyalgia [79]. Treatment of sleep disturbances leading to reductions of alpha sleep intrusions also results in lower levels of pain in these patients [61,62,76]. Although not a consensus in the literature, patients diagnosed with chronic fatigue syndrome also present reductions in sleep efficiency and REM sleep [58]. More recently, Orzeszek and colleagues showed no significant association between severity of pain and polysomnographic sleep parameters in patients with chronic orofacial pain, suggesting that this relationship does not appear to be present in all chronic pain conditions [28].

**Table 3:** Studies analyzing the impact of chronic pain on sleep.

Study	Design	Measurements	Sample	Level of Evidence
Diaz-Piedra et al. [79]	Systematic Review	PSQI, VAS, Likert-like scales, standardized questionnaires, PSG, actigraphy	34 studies totaling 3411 participants	High
Diaz-Piedra et al. [77]	Case-control	PSQI, ESS, HADS, MPQ, PSG	99 women	Moderate
Onen et al. [64]	RCT	VAS, MMSE, ESS, EPTh	11 participants	Low
Kallweit et al. [66]	Prospective Case Series	AHI, IHS criteria for migraine, VAS	11 participants	Low
Husak et al. [22]	Systematic Review	Multiple	64 studies	Moderate
Johnson et al. [67]	Cohort	PSG, ICHD-II criteria	82 participants	Low
Roberts et al. [74]	Cross-sectional	Berlin Sleep Questionnaire, PSQI, 7-item scale of the DSM-IV insomnia criteria, Sleep Disorders Questionnaire, DASS-21, CSQ, NRS	101 participants	Low
Goksan et al. [65]	Cross-sectional	PSG, ICHD-II, standardized questionnaire, HDS	563 participants	Low

AHI = Apnea-Hypopnea Index; CSQ = Coping Strategies Questionnaire; DASS-21 = Depression, Anxiety, and Stress Scales-21 item version; EPTh = Electrical Pain Threshold; ESS = Epworth Sleepiness Scale; HADS = Hospital Anxiety and Depression Scale; HDS = Hamilton Depression Scale; IHS = International Headache Society; ICHD-II= International Classification of Headache Disorders-2nd edition; ISI = Insomnia Severity Index; MMSE = Mini-Mental State Examination; MPQ = McGill Pain Questionnaire; NRS = Numerical Rating Scale; PSG = Polysomnography; PSQI = Pittsburgh Sleep Quality Index; RCT = Randomized Controlled Trial; VAS = Visual Analogue Scale.

## 6. Neural Mechanisms Underlying Pain and Sleep Disturbances

The relationship between pain and sleep is complex, with overlapping neural and biochemical pathways that are not yet fully understood. Various neurotransmitter systems, such as opioid, monoaminergic, and orexinergic pathways, contribute to both pain modulation and sleep regulation [80]. For instance, opioid receptors in brain regions like the preoptic area and periaqueductal gray are involved in controlling both pain and sleep [2,81]. Sleep deprivation disrupts the opioid and dopaminergic systems, both essential for pain regulation [82], leading to enhanced reactivity in the primary somatosensory cortex and diminished responses in higher-order brain regions involved in pain valuation, which could explain why sleep deprivation lowers pain thresholds, heightens pain experiences, and reduces the efficacy of opioid analgesics [2,81,83].

Recent research has highlighted additional brain regions, including the nucleus accumbens, lateral hypothalamic area, ventral tegmental area, supramammillary nucleus, dorsal raphe, and locus coeruleus, which have roles in both sleep and pain processes [84,85,86,87,88]. Notably, activation of dopamine receptor D1 neurons within a specific nucleus accumbens ensemble can simultaneously heighten nociceptive responses and reduce non-rapid eye movement sleep [87]. The ventral tegmental area, through its complex balance of glutamatergic and gamma-aminobutyric acid neuronal activity, influences both sleep–wake cycles and pain processing, modulating pain perception via dopaminergic pathways in the medial prefrontal cortex [84].

Recent research has also shown that circadian rhythms play substantial roles in modulating pain sensitivity [81,89]. For instance, pain sensitivity follows a daily cycle closely aligned with circadian rhythms, particularly at the spinal cord level, where structures such as the dorsal root ganglia are involved in pain signal transmission [89]. In addition, the suprachiasmatic nucleus, a key component of the circadian system, regulates pain perception by interacting with multiple brain regions, including the somatosensory cortices and insula, which are part of the “pain matrix” [90]. This suggests that the circadian system influences pain processing through its effects on different levels of the central nervous system, highlighting a shared regulatory network between pain and sleep.

These findings underscore the intricate neural circuitry and shared regulatory networks that govern pain and sleep, suggesting potential therapeutic targets for managing sleep disorders and chronic pain conditions through further research on these mechanisms.

## 7. Conclusions

Sleep disturbances and disorders are common and significant issues in chronic pain patients that can significantly impact their quality of life and pain outcomes. There is strong evidence that sleep disturbances are associated with increased pain severity, longer pain duration, greater levels of anxiety and depression, and impaired physical and psychosocial functioning in patients with chronic non-cancer pain. The data is less clear for patients with chronic cancer-related pain [17].

In addition, the literature largely concurs with a bidirectional relationship between chronic pain and sleep disturbances. Moreover, current evidence strongly suggests that sleep impairment is a stronger predictor of pain than pain is of sleep impairment [2,21,22,23,34]. However, the relative strength of each direction of the pain–sleep relationship remains uncertain. Therefore, further studies are needed to clarify the strength of the bidirectional relationship between different types of chronic pain and different types of sleep disturbances.

In agreement, the literature supports that treating sleep disturbances can decrease pain intensity in chronic pain patients. Furthermore, the treatment of comorbid sleep disturbance and sleep disorders in chronic pain patients has the potential to improve outcomes. Hence, addressing sleep disturbances in chronic pain patients is crucial, as poor sleep has also been linked to higher levels of disability, depression, and pain-related catastrophizing [22,23,26,91].

## Figures and Tables

**Table 1:** Studies analyzing the prevalence and types of sleep disturbances and sleep disorders in chronic pain patients.

Study	Design	Measurements	Sample	Level of Evidence
Sun et al. [19]	Systematic review and meta-analysis	PSQI, ISI	20 studies totaling 6175 participants	High
Mathias et al. [23]	Meta-analysis	PSG or clinical diagnosis	37 studies	High
Sancisi et al. [33]	Cross-sectional case-control	Diagnostic criteria based on ICSD-2 and ICHD-II	207 participants with chronic or episodic headache	Moderate
Silva et al. [26]	Systematic review	PSQI	14 studies totaling 19,170 participants	High
Burgess et al. [34]	Cross-sectional	MPQ, VAS, BDI, STAI, PANAS, CATS	99 participants with LBP	Low
Louie et al. [29]	Cross-sectional	Standardized questionnaire	23,134 participants	Low
Bigatti et al. [25]	Cohort	PSQI, MPQ, NRS, CES-D	600 participants with fibromyalgia	Moderate
Chung et al. [17]	Cross-sectional	ICD-10 codes	1,248,914 participants with cancer	Low
Vaughan et al. [20]	Cross-sectional	BPI, PSQI, PSQ-3, VSH	51 participants	Low
Orzeszek et al. [28]	Cross-sectional	PSG, MPQ, HIT-6, MIDAS, ISI, PSQI, ESS	114 participants	Low

BDI = The Beck Depression Inventory; BPI = Brief Pain Inventory; CATS = Pain Catastrophizing Scale; CES-D = Center for Epidemiologic Studies Depression Scale; ESS = Epworth Sleepiness Scale; HIT-6 = Headache Impact Test-6; ICHD-II = International Classification of Headache Disorders-2nd edition; ICSD-2 = International Classification of Sleep Disorders-2nd edition; ISI = Insomnia Severity Index; LBP = Low Back Pain; MIDAS = The Migraine Disability Assessment; MPQ = McGill Pain Questionnaire-Short Form; NRS = Numerical Rating Scale; PANAS = Positive and Negative Affect Schedule; PSG = Polysomnography; PSQ-3 = Pain and Sleep Questionnaire-3; PSQI = Pittsburgh Sleep Quality Index; STAI = State-Trait Anxiety Inventory; VAS = Visual Analog Pain Intensity Scale; VSH = Verran Snyder-Halpern Sleep Scale.
